# The Implications of the COVID-19 Pandemic on Trade

**DOI:** 10.1017/err.2020.48

**Published:** 2020-05-01

**Authors:** Ignacio CARREÑO, Tobias DOLLE, Lourdes MEDINA, Moritz BRANDENBURGER

**Affiliations:** * Senior Associate at FratiniVergano – European Lawyers, a law firm with offices in Brussels and Singapore that specialises in international trade and food law; email: i.carreno@fratinivergano.eu.; ** Senior Associate at FratiniVergano – European Lawyers.; *** Junior Associate at FratiniVergano – European Lawyers.; **** Junior Lawyer at FratiniVergano – European Lawyers.

## Abstract

On 17 March 2020, the President of the European Council, Charles Michel, and the President of the European Commission (hereinafter, Commission), Ursula von der Leyen, announced further European Union (EU) actions in response to the COVID-19 outbreak. Since the pandemic reached Europe, the EU has adopted a number of trade-related measures, including the issuance of guidelines for national border management, as well as export authorisation requirements. On 14 March 2020, the Commission adopted “Commission Implementing Regulation (EU) 2020/402 of 14 March 2020 making the exportation of certain products subject to the production of an export authorisation”, temporarily restricting exports of “personal protective equipment” to destinations outside of the EU. On 14 April 2020, the Commission announced that it would narrow down export authorisation requirements to protective masks only and extend the geographical and humanitarian exemptions. Governments around the world have been implementing trade-related measures in response to the COVID-19 pandemic, some trade restrictive, but a number of countries have also called for the elimination of export controls and restrictions on essential goods. As the greater implications of the COVID-19 pandemic on trade are still difficult to assess, the emergency measures taken by affected countries already require legal scrutiny. At the same time, it must be noted that, as noted above for the EU measures, measures around the world are subject to change dynamically in view of the evolution of the pandemic.

## I. Introduction

On 17 March 2020, the President of the European Council, Charles Michel, and the President of the European Commission (hereinafter, Commission), Ursula von der Leyen, announced further European Union (EU) actions in response to the COVID-19 outbreak.^1^ Since the pandemic reached Europe, the EU has adopted a number of trade-related measures, including the issuance of guidelines for national border management, as well as export authorisation requirements. On 14 March 2020, the Commission adopted “Commission Implementing Regulation (EU) 2020/402 of 14 March 2020 making the exportation of certain products subject to the production of an export authorisation”,^2^ temporarily restricting exports of “personal protective equipment” to destinations outside of the EU. On 14 April 2020, the Commission announced that it would narrow down export authorisation requirements to protective masks only and extend the geographical and humanitarian exemptions. Governments around the world have been implementing trade-related measures in response to the COVID-19 pandemic, some trade restrictive, but a number of countries have also called for the elimination of export controls and restrictions on essential goods. As the greater implications of the COVID-19 pandemic on trade are still difficult to assess, the emergency measures taken by affected countries already require legal scrutiny. At the same time, it must be noted that, as noted above for the EU measures, measures around the world are subject to change dynamically in view of the evolution of the pandemic.

## II. Measures introduced by the EU

In response to the COVID-19 pandemic, EU Member States endorsed “Guidelines for border management measures”^3^ in order to ensure the smooth passage of goods, particularly food, and medical and health supplies across EU Member States’ borders. President of the Commission von der Leyen stated that the measure would only be effective if there was coordination at the EU level. She pointed out that it was important to ensure that “goods and essential services continue to flow in our internal market” in order to prevent shortages of food and medical equipment.^4^ The guidelines set out “principles for an integrated approach to an effective border management to protect health while preserving the integrity of the Single Market”. Firstly, the guidelines recommend that, in relation to transport, EU Member States should impose transparent, science-based, proportionate, transport mode-specific and non-discriminatory measures. Furthermore, the measures should ensure unobstructed transport of essential goods, such as food and vital medical and health supplies. Secondly, EU Member States should preserve the free circulation within the EU Internal Market of all goods, particularly basic need products, such as medicines, medical equipment and food products. No additional certifications should be required for goods. Thirdly, EU Member States should designate priority “green lanes” at their borders for freight transport. In this regard, on 23 March 2020, the Commission published a “Communication from the Commission on the implementation of the Green Lanes under the Guidelines for border management measures to protect health and ensure the availability of goods and essential services”.^5^

On 14 March 2020, the Commission adopted “Commission Implementing Regulation (EU) 2020/402 of 14 March 2020 making the exportation of certain products subject to the production of an export authorisation”, temporarily restricting exports of “personal protective equipment” to destinations outside of the EU.^6^ This Implementing Regulation was adopted with the objective of ensuring the supply of “personal protective equipment” in the EU. Prior to the adoption of this implementing act, at the beginning of March 2020, certain EU Member States, such as France and Germany, had already imposed restrictions on exports of protective medical equipment, such as face masks.^7^ As these measures also applied to other EU Member States, they caused considerable disruption within the internal market and irritation among EU Member States. These national measures were removed when Regulation (EU) 2020/402 entered into force. Implementing Regulation (EU) 2020/402 introduced an export authorisation requirement for certain specified “personal protective equipment” defined in Annex I of the Regulation and including, notably, protective spectacles and visors, face shields, mouth–nose protection equipment, protective garments and gloves. According to Article 3 of Regulation (EU) 2020/402, the Regulation is to “apply for a period of six weeks” and will “automatically cease to apply at the end of this six weeks period”. Businesses intending to export any such products are required to apply for an export authorisation with the competent EU Member States’ authorities. Before granting an export authorisation, Article 2(3) Regulation (EU) 2020/402 requires the competent authority to consider the export objective in order to allow, for instance, coordinated EU efforts against COVID-19. On 19 March 2020, the Commission amended Regulation (EU) 2020/402, exempting Andorra, the Faroe Islands, Liechtenstein, Norway, San Marino, Switzerland and the Vatican, as well as certain overseas territories, from the export restriction.^8^ The Commission adopted Regulation (EU) 2020/402 and the amendment thereto via an urgency procedure in accordance with Article 5 of “Regulation (EU) 2015/479 of the European Parliament and of the Council on common rules for exports”. On 20 March 2020, the Commission published a “Guidance note to Member States related to Commission Implementing Regulation (EU) 2020/402 making the exportation of certain products subject to the production of an export authorisation, as last amended by Commission Implementing Regulation (EU) 2020/426”,^9^ providing certain clarifications, including that exports to the UK would not be considered as exports to a third country. The Commission underlined that the measure taken by the EU was a temporary export restriction and not an export ban and that it was in line with the EU’s international obligations. On 14 April 2020, the Commission announced that it had started consultations with EU Member States on a draft regulation to adjust the export authorisation scheme set up by Implementing Regulation 2020/402, which elapsed on 25 April 2020. Through “Commission Implementing Regulation (EU) 2020/568 of 23 April 2020 making the exportation of certain products subject to the production of an export authorisation”, the Commission narrowed down the export authorisation requirements to protective masks only and extended the geographical and humanitarian exemptions to the Western Balkans, as well as to Gibraltar and territories of EU Member States excluded from the EU Customs Union.^10^ The new regulation applies since 26 April 2020 and is again limited to a period of 30 days. According to the Commission, the category of protective masks is the only category for which an export authorisation is necessary in order to secure adequate supply to protect the health of EU citizens. In the spirit of international solidarity, Article 2(6) of Implementing Regulation (EU) 2020/568 explicitly requires EU Member States to authorise exports of emergency supplies in the context of humanitarian aid and to process the relevant applications in an expedite manner.

In addition, the EU also decided to suspend tariffs and value-added tax (VAT) on specific goods under strict conditions and, on 3 April 2020, published “Commission Decision on relief from import duties and VAT exemption on importation granted for goods needed to combat the effects of the COVID-19 outbreak during 2020”. On 30 March 2020, the EU published a “Guidance on Customs issues related to the COVID-19 emergency”.^11^ The Guidance, *inter alia*, explains the temporary suspension of certain import tariffs and explains that temporary admission procedures had been implemented for goods brought into the customs territory of the EU to counter the effects of COVID-19 in accordance with Article 141(1)(d) of “Commission Delegated Regulation (EU) 2015/2446 of 28 July 2015 supplementing Regulation (EU) No 952/2013 of the European Parliament and of the Council as regards detailed rules concerning certain provisions of the Union Customs Code” (ie the Union Customs Code Delegated Act).^12^ These goods are supposed to be temporarily admitted duty-free.

## III. Export restrictions adopted by countries around the world

Similar export restrictions have been adopted by governments around the world as countries respond to the COVID-19 pandemic. For instance, on 18 March 2020, Indonesia’s Ministry of Trade announced a temporary export ban on face masks, sanitisers and certain medical equipment until 30 June 2020.^13^ India has also banned exports of similar products, as well as of all types of ventilators and anti-malarial medicines.^14^ Importantly, it appears that such export restrictions are already causing a shortage of such products in certain countries looking to purchase them. Adrian van den Hoven, the Director General of Medicines for Europe, a trade association representing the European generic, biosimilar and valued-added pharmaceutical industries, noted on 22 March 2020 that hospitals in Romania, at that time, lacked sufficient intravenous medicines, which are normally manufactured in Serbia and exported to Romania.^15^ However, as Serbia had closed its borders and restricted exports, the shipments were currently unavailable.^16^ Serbia is an important manufacturing centre not only for Romania, but also for other countries, such as Germany.

On 22 March 2020, Kazakhstan, one of the world’s most important exporters of wheat flour, banned all exports thereof, and the ban also extends to carrots, sugar and potatoes.^17^ Serbia has banned, *inter alia*, exports of sunflower oil, and, on 25 March 2020, the Prime Minister of Vietnam, Nguyen Xuan Phuc, announced that all new rice export contracts would be suspended until a report on the country’s rice stocks was available.^18^ Other countries have also taken export restrictions on food. For instance, El Salvador and Honduras adopted a temporary export ban on certain dried leguminous vegetables, such as red beans, in order to guarantee domestic supplies.^19^ Kyrgyzstan implemented restrictions on certain food products, such as wheat and meslin (ie a mixture of cereal species that are sown and harvested together), wheat flour, cooking oil, rice, pasta, chicken eggs, sugar and iodised table salt.^20^

On 9 April 2020, the Government of EU Member State Romania issued an export ban of wheat, corn, rice, sunflower seeds and other grains, vegetable oils, sugar and various bakery products. While the measure provided that food products were allowed to be sold within the EU, buyers had to prove that purchased products were not intended for export outside of the EU. The European Commissioner for Agriculture and Rural Development, Janusz Wojciechowski, declared that the Commission did “not have any information, which indicates that Romania is facing or will soon face shortages of agricultural products intended for human consumption” and that the reported measure appeared “to be not proportionate”.^21^ Therefore, and following an assessment that found that the country’s food security was currently not under threat, the export ban was revoked on 16 April 2020.^22^

The impact of the increasing number of trade-related measures as part of government responses to the COVID-19 pandemic are poised to significantly disrupt the global trading system, supply chains and trade flows. A shortage of certain products could also lead to higher prices and even endanger food security in certain countries.

## IV. COVID-19-related export restrictions and their compliance with World Trade Organization rules

Export restrictions are generally prohibited by the World Trade Organization (hereinafter, WTO). Exceptions to this rule are only allowed in specific circumstances under Articles XI:2, XX and XXI(b) of the General Agreement on Tariffs and Trade (hereinafter, GATT). Article XI:2 of the GATT expressly allows “export prohibitions or restrictions temporarily applied to prevent or relieve critical shortages of foodstuffs or other products essential to the exporting contracting party”. Article XX(b) of the GATT allows measures “necessary to protect human, animal or plant life or health”. Furthermore, Article XXI(b)(iii) on “Security exceptions” states that nothing in the GATT must be construed to prevent any WTO Member “from taking any action which it considers necessary for the protection of its essential security interests” in times of “emergency in international relations”.

In view of previous WTO Appellate Body jurisprudence, it appears that the current measures may be justified on the grounds of Article XI:2 of the GATT as *temporary* measures in view of *critical shortages* of *essential goods*. In the case *China – Raw Materials*,^23^ the Appellate Body states that it considers “that Article XI:2(a) describes measures applied for a limited duration, adopted in order to bridge a passing need, irrespective of whether or not the temporal scope of the measure is fixed in advance”.^24^ A critical shortage is defined as “deficiencies in quantity that are crucial, that amount to a situation of decisive importance, or that reach a vitally important or decisive stage, or a turning point”.^25^ The term “essential” is defined as “[a]bsolutely indispensable or necessary”.^26^ Considering the current crisis, the WTO Appellate Body’s interpretation of Article XI:2 of the GATT appears to provide WTO Members with the authority to restrict exports of food and medical supplies as long as necessary in order to prevent critical shortages.^27^

Regarding medical products, WTO Members exporting such products may also invoke the general exception of Article XX(b) of the GATT. Article XX(b) of the GATT allows measures necessary to protect human life or health, provided that these measures “are not applied in a manner which would constitute a means of arbitrary or unjustifiable discrimination between countries where the same conditions prevail, or a disguised restriction on international trade”. Taking these requirements into account, it appears that undiscriminating export restrictions on medical equipment are in accordance with WTO law, as long as they are strictly limited to the requirements of emergency response.^28^

However, the above-cited Article XXI(b) of the GATT regarding the protection of security interests in times of an emergency in international relations does not appear to be a suitable justification for the current measures. Even though the applicability of Article XXI(b) of the GATT in the context of the COVID-19 pandemic is discussed,^29^ the WTO Appellate Body typically applies a State-centred definition of “emergency in international relations”, which revolves around armed conflicts and does not include health emergencies.^30^

## V. Safeguarding global trade

On 25 March 2020, Australia, Brunei, Canada, Chile, Myanmar, New Zealand and Singapore issued a joint statement urging countries to “ensure that trade lines remain open, including via air and sea freight, to facilitate the flow of goods including essential supplies”.^31^ The countries underlined the importance of removing restrictions on essential goods and medical supplies, that they were committed “to working with all like-minded countries to ensure that trade continues to flow unimpeded”, and that critical infrastructure, such as airports and seaports, remained open “to support the viability and integrity of supply chains globally”.

Already on 24 March 2020, WTO Director-General Roberto Azevêdo had requested WTO Members to submit information to the WTO Secretariat regarding policies that they had introduced in response to the COVID-19 outbreak. Director-General Azevêdo pointed out the importance of transparency with regards to trade-related measures and argued that an overview of the measures taken by WTO Members “would be particularly useful for the many countries that rely on imports for medical supplies”. Additionally, Director-General Azevêdo created a task force of experts from across the WTO Secretariat to monitor the impacts of COVID-19 on trade flows and the overall global economy. On 3 April 2020, the WTO published a report on “Trade in Medical Goods in the Context of Tackling Covid-19”.^32^ The report provides an overview of trade and tariffs imposed on medical goods, considered as relevant in the context of COVID-19. The report categorised the medical products into four groups: (1) medicines (ie pharmaceuticals); (2) medical supplies (eg alcohol, syringes and gauze); (3) medical equipment and technology; and (4) personal protective products (eg hand soap and sanitiser, face masks and protective spectacles).^33^ Overall, the report notes that tariffs on medical products are considerably low. The WTO has contributed to the liberalisation of trade in these products by means of: (1) the tariff schedules agreed at the time of establishment of the WTO in 1995; (2) the conclusion of the Plurilateral Sectoral Agreement on Pharmaceutical Products; and (3) the expansion of the Information Technology Agreement (ITA) in 2015, which covers certain medical equipment.^34^ However, the report also notes that an average of 11.5% tariff is applied on COVID-19-relevant personal protective products, such as hand soap and sanitiser, hand gloves, face masks and protective spectacles, which is partly attributed to the fact that most of these products are not included in any of the aforementioned arrangements.

In recent weeks, multilateral fora have been increasingly advocating for global action to safeguard global trade. According to WTO Deputy Director-General Wolff, a discussion paper of 11 March 2020 for a meeting of the so-called Ottawa Group of WTO Members focused on WTO reforms and highlighted two topics of discussion: (1) tariff reductions as a fiscal stimulus measure; and (2) tariff eliminations as a direct response to the COVID-19 pandemic. WTO Deputy Director-General Wolff underlined that there was “neither sufficient time nor evident WTO Member desire to initiate and conclude a new round of tariff negotiations”, and that the “only effective step would be to engage in autonomous coordinated tariff reduction”. Such tariff elimination should be reciprocal, with other WTO Members voluntarily replicating the approach. The WTO has also called on the Group of Twenty (hereinafter, G20), an international forum of the governments and central bank governors from 19 countries and the EU, to increase international cooperation. In a joint statement, countries in the G20 stated that they remained committed to international cooperation and to working together to facilitate international trade and to coordinate their responses.^35^ On 6 April 2020, the World Customs Organization (hereinafter, WCO) and the WTO issued a joint statement, noting that the WCO was willing “to establish a coordinated approach in support of initiatives that facilitate cross-border trade in goods, in particular those key to combat COVID-19”.^36^ To respond to the unprecedented worldwide demand in medical supplies amid the current global COVID-19 pandemic, and in order to assist countries in facilitating the cross-border movement of these critical products, on 9 April 2020, the WCO and the World Health Organization (hereinafter, WHO) updated the “HS Classification Reference for COVID-19 Medical Supplies” to a more structured and user-friendly format in order to reflect more of the products required in public health.^37^ Furthermore, the WHO, the Food and Agriculture Organization of the United Nations and the WTO issued a joint statement noting that millions of people worldwide depended on international trade for their food security or livelihoods, and that countries should ensure that trade-related measures do not disrupt food supply chains.^38^

In order to ensure transparency, the WTO put in place a webpage dedicated to COVID-19, providing up-to-date trade-related information and an updated list of the various notified measures adopted by WTO Members.^39^ Furthermore, WTO Deputy Director-General Wolff stated that the WTO could be an essential forum for international cooperation to ensure that trade policies contribute to respond to the current crisis and reduce the severe negative impact on the world economy of the COVID-19 pandemic.

## VI. Conclusion

The COVID-19 pandemic will likely take months to overcome, and the impact on trade will be significant and lasting. The situation is changing dynamically and rapidly, which also applies to the measures and regulations discussed in this article. However, it can be expected that, subject to the slowdown of the pandemic and an increased understanding of its dynamics, countries will make efforts to reopen their markets in accordance with their WTO obligations to restrict trade to the least possible extent. As has already been seen, trade-restrictive measures are adopted, amended and revoked on a daily basis. Therefore, traders around the world must closely monitor the trade-related developments linked to the COVID-19 pandemic and should remain up to date on the measures in force.

## Footnotes

1 Video conference of the members of the European Council, 17 March 2020 <https://www.consilium.europa.eu/en/meetings/european-council/2020/03/17> (last accessed 9 April 2020).

2 Commission Implementing Regulation (EU) 2020/402 of 14 March 2020 making the exportation of certain products subject to the production of an export authorisation, OJ L 77I, 15.3.2020, pp 1–7 <https://eur-lex.europa.eu/legal-content/GA/TXT/?uri=CELEX:32020R0402> (last accessed 9 April 2020).

3 Covid-19 Guidelines for border management measures to protect health and ensure the availability of goods and essential services 2020, OJ C 86 I/01, 16.3.2020, pp 1–4 <https://eur-lex.europa.eu/legal-content/EN/TXT/?uri=uriserv:OJ.CI.2020.086.01.0001.01.ENG&toc=OJ:C:2020:086I:TOC> (last accessed 9 April 2020).

4 COVID-19: Commission presents guidelines for border measures to protect health and keep goods and essential services available, European Commission, 16 March 2020 <https://ec.europa.eu/commission/presscorner/detail/en/IP_20_468> (last accessed 9 April 2020).

5 Communication from the Commission on the implementation of the Green Lanes under the Guidelines for border management measures to protect health and ensure the availability of goods and essential services 2020/C 96 I/01, OJ C 96I, 24.3.2020, pp 1–7 <https://eur-lex.europa.eu/legal-content/EN/TXT/?uri=uriserv:OJ.CI.2020.096.01.0001.01.ENG&WT.mc_id=Twitter> (last accessed 9 April 2020).

6 Commission Implementing Regulation (EU) 2020/402 of 14 March 2020 making the exportation of certain products subject to the production of an export authorisation, OJ L 77I, 15.3.2020, pp 1–7 <https://eur-lex.europa.eu/legal-content/GA/TXT/?uri=CELEX:32020R0402> (last accessed 9 April 2020). On this issue, see: I van Damme, “European Union Imposes Export Restrictions on Personal Protective Equipment”, *EU Law Live*, 17 March 2020 <https://eulawlive.com/analysis-european-union-imposes-export-restrictions-on-personal-protective-equipment-by-isabelle-van-damme> (last accessed 20 April 2020); L Arroyo Jiménez and M Eliantonio, “Masks, Gloves, Exports Licences and Composite Procedures: Implementing Regulation 2020/402 and the Limelight of Accountability” (2020) European Journal of Risk Regulation DOI: 10.1017/err.2020.28.

7 “E.U. Seeks Solidarity as Nations Restrict Medical Exports”, *New York Times*, 7 March 2020 <https://www.nytimes.com/2020/03/07/business/eu-exports-medical-equipment.html> (last accessed 9 April 2020).

8 Commission Implementing Regulation (EU) 2020/426 of 19 March 2020 amending Implementing Regulation (EU) 2020/402 making the exportation of certain products subject to the production of an export authorization C/2020/1864, OJ L 84I, 20.3.2020 <https://eur-lex.europa.eu/legal-content/EN/TXT/?uri=uriserv:OJ.LI.2020.084.01.0001.01.ENG&toc=OJ:L:2020:084I:TOC> (last accessed 20 April 2020).

9 Communication from the Commission Guidance note to Member States related to Commission Implementing Regulation (EU) 2020/402 making the exportation of certain products subject to the production of an export authorisation, as last amended by Commission Implementing Regulation (EU) 2020/426 2020/C 91 I/02, OJ C 91I, 20.3.2020, pp 10–15 <https://eur-lex.europa.eu/legal-content/EN/TXT/?uri=uriserv:OJ.CI.2020.091.01.0010.01.ENG&toc=OJ:C:2020:091I:TOC> (last accessed 9 April 2020).

10 Commission Implementing Regulation (EU) 2020/568 of 23 April 2020 making the exportation of certain products subject to the production of an export authorisation, OJ L 129, 24.4.2020, pp 1–7 <https://eur-lex.europa.eu/legal-content/EN/ALL/?uri=uriserv:OJ.L_.2020.129.01.0007.01.ENG> (last accessed 1 May 2020).

11 Guidance on Customs issues related to the COVID-19 emergency, European Commission (March 2020) <https://ec.europa.eu/taxation_customs/sites/taxation/files/covid-19-customs-guidance-for-trade.pdf> (last accessed 14 April 2020).

12 Commission Delegated Regulation (EU) 2015/2446 of 28 July 2015 supplementing Regulation (EU) No 952/2013 of the European Parliament and of the Council as regards detailed rules concerning certain provisions of the Union Customs Code, OJ L 343, 29.12.2015, pp 1–557 <https://eur-lex.europa.eu/legal-content/EN/ALL/?uri=CELEX:32015R2446> (last accessed 20 April 2020).

13 “Indonesia to Ban Face Mask Exports to Ensure Domestic Supply”, *Reuters*, 13 March 2020 <https://www.reuters.com/article/us-health-coronavirus-indonesia-masks/indonesia-to-ban-face-mask-exports-to-ensure-domestic-supply-idUSKBN2100JB> (last accessed 20 April 2020).

14 “WTO Asks Members to Share Information on Trade Measures Related to COVID-19”, *Financial Express*, 25 March 2020 <https://www.financialexpress.com/economy/wto-asks-members-to-share-information-on-trade-measures-related-to-covid-19/1909190> (last accessed 14 April 2020).

15 I Dreyer, “EU External Border and Air Freight: Next Medical Supply Chain Flash Point?”, *Borderlex*, 24 March 2020 <https://borderlex.eu/2020/03/24/eu-external-border-and-air-freight-next-medical-supply-chain-flash-point> (last accessed 20 April 2020).

16
ibid.

17 “Countries Starting to Hoard Food, Threatening Global Trade”, *Bloomberg*, 25 March 2020 <https://finance.yahoo.com/news/countries-starting-hoard-food-threatening-233000095.html> (last accessed 14 April 2020).

18
ibid.

19 WTO Members’ notifications on COVID-19, WTO (April 2020) <https://www.wto.org/english/tratop_e/covid19_e/covid_measures_e.pdf> (last accessed 14 April 2020)

20
ibid.

21 “EU Objects to Romania’s Move to Ban Agriculture Exports”, *Bloomberg*, 11 April 2020 <https://www.bloomberg.com/news/articles/2020-04-11/eu-objects-to-romania-s-move-to-ban-agriculture-exports> (last accessed 14 April 2020).

22 “Romania Lifts All Wheat Export Restrictions – Interior Minister”, *Reuters*, 16 April 2020 <https://www.reuters.com/article/health-coronavirus-wheat-romania-idUSL5N2C45XQ> (last accessed 17 April 2020).

23 WTO Appellate Body Report on *China – Raw Materials* of 30 January 2012, WT/DS394/AB/R; WT/DS395/AB/R; WT/DS398/AB/R.

24
ibid, para 331.

25
ibid, para 324.

26
ibid, para 326.

27 See J Pauwelyn, “Export Restrictions in Times of Pandemic: Options and Limits under International Trade Agreements”, p 6 f. <https://papers.ssrn.com/sol3/papers.cfm?abstract_id=3579965> (last accessed 20 April 2020).

28
ibid, p 8 f.

29 See, eg, V Lapa, “GATT Article XXI as a Way to Justify Food Trade Restrictions Adopted as a Response to COVID-19?” <http://regulatingforglobalization.com/2020/04/10/gatt-article-xxi-as-a-way-to-justify-food-trade-restrictions-adopted-as-a-response-to-covid-19/?doing_wp_cron=1587047010.5144829750061035156250> (last accessed 20 April 2020).

30 WTO Panel Report of 5 April 2019, WT/DS512/R, para 7.76.

31 Joint Ministerial Statement by Australia, Brunei Darussalam, Canada, Chile, the Republic of the Union of Myanmar, New Zealand and Singapore affirming commitment to ensuring supply chain connectivity amidst the COVID-19 situation, Government of Canada, 25 March 2020 <https://www.international.gc.ca/gac-amc/news-nouvelles/2020-03-25-joint-ministerial-statement-declaration-ministerielle-commune.aspx?lang=eng> (last accessed 14 April 2020).

32 “Trade in Medical Goods in the Context of Tackling Covid-19”, WTO, 3 April 2020, <https://www.wto.org/english/news_e/news20_e/rese_03apr20_e.pdf> (last accessed 14 April 2020).

33
ibid.

34
ibid.

35 “Extraordinary G20 Leaders’ Summit Statement on COVID-19”, G20, March 2020 <https://g20.org/en/media/Documents/G20_Extraordinary%20G20%20Leaders%E2%80%99%20Summit_Statement_EN%20(3).pdf> (last accessed 14 April 2020).

36 “WCO–WTO Joint Statement on COVID-19 Related Trade Measures”, WCO, 6 April 2020 <http://www.wcoomd.org/en/media/newsroom/2020/april/wco-wto-joint-statement-on-covid-19-related-trade-measures.aspx> (last accessed 14 April 2020).

37 “Joint WCO/WHO HS Classification List for COVID-19 Medical Supplies Issued” <http://www.wcoomd.org/en/media/newsroom/2020/april/joint-wco-who-hs-classification-list-for-covid_19-medical-supplies-issued.aspx> (last accessed 16 April 2020).

38 “Mitigating Impacts of COVID-19 on Food Trade and Markets”, FAO, 31 March 2020 <http://www.fao.org/news/story/en/item/1268719/icode> (last accessed 14 April 2020).

39 “COVID-19 and World Trade” <https://www.wto.org/english/tratop_e/covid19_e/covid19_e.htm> (last accessed 16 April 2020).

